# Hand Movement Classification Using Burg Reflection Coefficients

**DOI:** 10.3390/s19030475

**Published:** 2019-01-24

**Authors:** Daniel Ramírez-Martínez, Mariel Alfaro-Ponce, Oleksiy Pogrebnyak, Mario Aldape-Pérez, Amadeo-José Argüelles-Cruz

**Affiliations:** 1 Centro de Investigación en Computación, Instituto Politécnico Nacional, Av. “Juan de Dios Bátiz” s/n esq. Miguel Othón de Mendizábal, Col. Nueva Industrial Vallejo, Del. Gustavo A. Madero, Ciudad de México C.P. 07738, Mexico; dhanielrhamirez@gmail.com (D.R.-M.); oleksiy@cic.ipn.mx (O.P.); 2 Departamento de Ciencias e Ingenierías, Universidad Iberoamericana Puebla, Blvrd del Niño Poblano 2901, Reserva Territorial Atlixcáyotl, Centro Comercial Puebla, San Andrés Cholula 72810, Puebla, Mexico; marielalfa@gmail.com; 3 Centro de Innovación y Desarrollo Tecnológico en Cómputo, Instituto Politécnico Nacional, Av. “Juan de Dios Bátiz” s/n esq. Miguel Othón de Mendizábal, Col. Nueva Industrial Vallejo, Del. Gustavo A. Madero, Ciudad de México C.P. 07700, Mexico; maldape@ipn.mx

**Keywords:** electromyography, hand movement, health monitoring, maximum entropy reflection coefficients, classification algorithms, machine learning, feature selection

## Abstract

Classification of electromyographic signals has a wide range of applications, from clinical diagnosis of different muscular diseases to biomedical engineering, where their use as input for the control of prosthetic devices has become a hot topic of research. The challenge of classifying these signals relies on the accuracy of the proposed algorithm and the possibility of its implementation in hardware. This paper considers the problem of electromyography signal classification, solved with the proposed signal processing and feature extraction stages, with the focus lying on the signal model and time domain characteristics for better classification accuracy. The proposal considers a simple preprocessing technique that produces signals suitable for feature extraction and the Burg reflection coefficients to form learning and classification patterns. These coefficients yield a competitive classification rate compared to the time domain features used. Sometimes, the feature extraction from electromyographic signals has shown that the procedure can omit less useful traits for machine learning models. Using feature selection algorithms provides a higher classification performance with as few traits as possible. The algorithms achieved a high classification rate up to 100% with low pattern dimensionality, with other kinds of uncorrelated attributes for hand movement identification.

## 1. Introduction

Electromyography (EMG) is an electrodiagnostic medical procedure to assess the health of muscles and the nerve cells that control them, with the detection, recording, and analysis of surface electromyography signals (sEMG) [1]. EMG provides physicians and health experts with the information generated by the muscle contractions, that is the ionic flow through the muscle fiber [2]. Research has considered EMG as an important field of study due to the diversity of its applications in clinical medicine and biomedical engineering [3]. EMG has applications such as the diagnosis of nervous system disorders and muscular diseases like myopathy and neuropathies [4,5,6]. All these applications require the preprocessing of the signals and the extraction of their features [7]. sEMG are useful as input control signals for prosthetic limbs [8,9], in rehabilitation as a measurement parameter of muscular effort [10], and for the development of muscle machine interfaces [11].

Most EMG applications involve real-time systems, which need to run with low-cost computational features [12]. As a matter of fact, in the development of prosthetic, orthotic, and rehabilitation devices, EMG can be employed as a part of the control system [13]. In the results reported by [14], EMG pattern recognition and myoelectric control were compared for the control of prosthetics, and the paper remarked that these signals were suitable for the control, highlighting the implementations of algorithms that were capable of distinguishing between signals that had similarities. These similarities presented in users that had lost a body part, as a consequence of the absence of peripheral structures in the musculoskeletal system, where the classification of EMG features becomes a challenge [15]. EMGs features are listed on Table 1, these characteristics have been used in different classification tasks [16,17,18,19,20], and the classification rate increases with the use of a proper signal preprocessing stage; for instance, Chowdhury et al. considered the use of wavelet and empirical mode decomposition, first differentiation, or independent component analysis [19]. Despite the performances achieved at the preprocessing stage, the computational complexity might increase, adding a delay to the response.

Several authors used autoregressive models and the characteristics of random processes, such as first and second moments, etc., in tasks related to the classification of myopathy or neuropathy [21]. For example, Bozkurt et al. reported a 97% classification performance using fifteenth order AR models, Yule–Walker, Burg, covariance, modified covariance, and subspace-based methods to extract features from 1200 sEMG, applying high-resolution and a high-sampling rate invasive electrodes implanted in a bicep brachii muscle [22].

In a different research work dedicated to hand movement, Phinyomark et al. reported a high classification rate of 97.76%, achieved by applying a quadratic discriminant analysis and four AR coefficients per channel, including a preprocessing stage whose output was the first differentiation of sEMG [23]. They extracted information from the activity of five forearm muscles features: WL, DAMV, DASDV, DVARV, DASDV, M2, WAMP, IEMG, and MAV. They also used these features in their previous works [24]. The features SSI, VAR, RMS, MYOP, CC, LOG, TK, and V from another point of view were used in [23], applying the seventh order Daubechies mother wavelet and the four decomposition levels before sEMG characterization to extract RMS and MAV. They tested the behavior of these features to estimate whether they were useful for identification of six daily hand movements, while monitoring flexor and extensor carpi radialis longus muscles.

Liu et al. described the use of a support vector machine (SVM) ensemble [25] to classify eight different hand grasps with a precision rate of 93.54%; extracting sEMG from three different forearm muscles, the fourth order AR coefficients and the histogram of EMG (HEMG) allowed building the feature vector per channel. In their work, the aim was to get significant features and a classification model that permitted increasing the classification rate of sEMG. However, the SVM ensemble used resulted in being computationally expensive [26]. Angari et al. considered fifteen channels to digitalize sEMG and to characterize five hand movements, where they extracted twenty-one attributes per channel (MAV, WL, ZC, SSC, AR, among others) to implement feature selection methods and perform channel discrimination [27]. In this case, the aim of the research was to train the SVM with low dimensionality patterns and the most representative forearm muscles; this work concluded that MAV and WL were appropriate for classification tasks.

The method of Khezri et al. used an adaptive neuro-fuzzy inference system to test its classification rate in a six-hand movement dataset containing four channels [28]. The considered features were MAV, SSC, ZC, and 10 order AR model coefficients. Merging these attributes to create patterns for sEMG representation resulted in classification rates of 86–100%.

Ruangpaisarn et al. presented a feature extraction technique for hand movement classification, considering two pairs of EMG electrodes and the merging and transformation of both channels into a squared matrix to perform factorization via singular-value decomposition [29]. They reported the use of singular values in the matrix’s main diagonal and the training of SVM with fifty feature instances, achieving a performance of 98.22%. The issue in this work was comprised of taking samples, where no muscular activity was investigated, and working with a 2D vector in most cases led to non-linear computational complexity. With the same dataset, Sapsanis et al. used a preprocessing stage in which signals were decomposed into three levels with empirical mode decomposition, so that the noise was reduced [30]. For each decomposition level and raw sEMG, they extracted the following attributes: IEMG, ZC, VAR, SSC, WL, WAMP, kurtosis, and skewness. With linear discrimination analysis, the rate of correct classifications reached 89.21%.

Zhai et al. suggests a self-recalibrating classifier for hand movement, based on a convolutional neural network [31], where the algorithm’s update has the potential to keep a stable behaviour with no user retraining. Reviews in [18,19] points up the variety of features needed for classifying sEMG and preprocessing approaches that might lead to improvement of the model’s performance. Table 2 presents the classification accuracy achieved by different algorithms that worked with the EMG database from the University of California at Irvine (UCI) machine learning repository, where none of the studies adopted the reflection coefficients as features for pattern recognition.

The aim of this work is to develop a classification algorithm for sEMG with low computational cost and with a competitive classification rate. The remainder of this paper is presented as follows: Section 2 describes the hand movement database that was employed, as well as a brief review of the signal preprocessing techniques, and different features useful for classification are described. Then, the proposed classification method is presented. Section 3 shows the results obtained by the classification technique. Section 4 and Section 5 are the discussion and conclusion of the results achieved by the proposed methodology for sEMG classification.

## 2. Materials and Methods

This section provides a complete description of the dataset of hand movements used for the training and validation of the proposed algorithm, as well as the mathematical background for the preprocessing of the signal and its classification by the Burg reflection coefficients.

### 2.1. Data Selection and Preprocessing

We used an EMG dataset from the University of California at Irvine (UCI) machine learning repository, the same as in [29,30]. The data describe six different hand movements taken from the flexor carpi ulnaris and extensor carpi radialis muscles of five healthy people (three women and two men) who performed each hand action thirty times with no restrictions for 6 seconds each; the signal sampling frequency was 500 Hz. The dataset contains 1800 time series available to classify 6 hand grasps (spherical, tip, palmar, lateral, cylindrical, and hook).

As described in [30], EMG data were collected using two Delsys DE-2.1 EMG bar sensors made out of silver (Au) with a 10-mm inter-electrode distance [33]. Similar sensors were used in [34] to conduct a cross-talk analysis based on electrode spacing, and it was found that a 10-mm distance offered less cross-talk signal contamination. The sensor placement and orientation were guided by the specifications found in [33,35]. The inter-electrode distance matched the guidelines found in [36], which recommends a 20-mm distance and, in the case of relatively small muscles, an inter-distance less than or equal to a quarter of the muscle length. The main factors that introduce cross-talk to EMG data are related to sensor features such as physical dimension and shape, placement and orientation with respect to the muscle fibers, and inter-electrode distance. Since these factors were considered in the data acquisition process, the presence of cross-talk was considered minimal.

**Figure 1:**
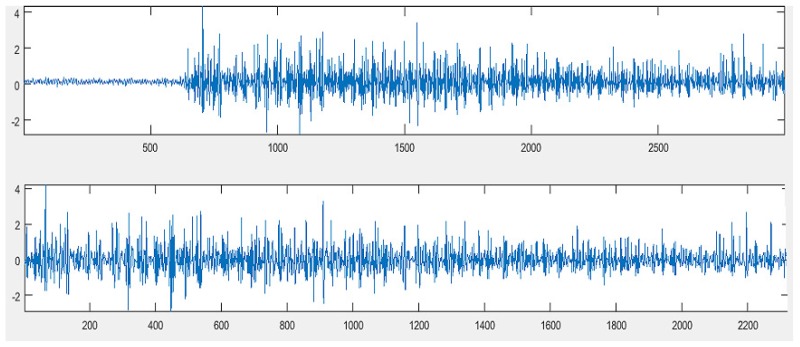
On top, the original signal; on the bottom, the clipped signal with a zero mean value.

Before feature extraction, a simple preprocessing treatment was applied to each signal. In the first preprocessing stage, the method eliminated the initial samples, where muscle activation was absent and only noise was present, so that the feature extraction did not lack the information about the phenomena. The next step comprised the extraction of the signal mean value at all data points; this operation is important to comply with the restrictions imposed by the optimal linear filtering theory [37]. The linear prediction model framework requires restrictions such as for autoregressive models. Otherwise, the performance of the classification/prediction models might decrease. Here, we used the simple arithmetic mean value computed as:

(1) $$\bar{x}=\frac{1}{N}\sum_{\begin{array}{c} n=0 \end{array}}^{\begin{array}{c} N \end{array}}\begin{array}{c} x[n] \end{array}$$

where $\bar{x}$ is the mean value; x[n] is the EMG signal, and *N* is the total number of samples. The application of the mean (1) implies a new sample value, which is described as $\bar{x}\left[n\right]=x\left[n\right]-\bar{x}$ for 1≤n≤N. Figure 1 illustrates the proposed preprocessing stages.

These two conditioning steps have a linear complexity and supply the feature extraction stage with an appropriate sEMG.

### 2.2. Standard Time Domain Features

The integrated EMG feature is defined as the cumulative addition of each signal sample absolute value:

(2) $$\mathrm{IEMG}=\sum_{\begin{array}{c} n=0 \end{array}}^{\begin{array}{c} N \end{array}}\left|x_{n}\right|$$

where the other attribute is the mean absolute value; it is one of the most useful attributes in many research works and consists of computing the mean absolute amplitude value of sEMG:

(3) $$\mathrm{MAV}=\frac{1}{N}\sum_{n=0}^{N}\left|x_{n}\right|$$

The simple squared integration feature describes the energy of sEMG and is mathematically defined as cumulative addition of the absolute squared value of each sample:

(4) $$\mathrm{SSI}=\sum_{n=1}^{N}{\left|x_{n}\right|}^{2}$$

A stochastic process, such as sEMG, can be defined by its first and second order moments, i.e., the mean and variance values. Therefore, these features might be part of the pattern. The mathematical definition for the variance considers that sEMG is a near to zero mean process, so its definition becomes:

(5) $$\mathrm{VAR}=\frac{1}{N-1}\sum_{n=1}^{N}{x_{n}}^{2}$$

The root mean squared value (RMS) reveals the information about the amount of strength yielded by a muscle and is defined as the square root of the mean squared values. In many research works, this attribute is considered important for different tasks:

(6) $$\mathrm{RMS}=\sqrt{\frac{1}{N}\sum_{n=1}^{N}{x_{n}}^{2}}$$

The wavelength is the distance between a pair of adjacent samples along all sEMG:

(7) $$\mathrm{WL}=\sum_{n=1}^{N}\left|x_{n+1}-x_{n}\right|$$

The zero-crossing feature describes the number of times that the sEMG amplitude becomes positive or negative. Its definition considers a threshold, whose aim is to count only the events produced by muscular activity:

(8) $$\mathrm{ZC}=\sum_{n=1}^{N-1}\left[sgn\left(x_{n}\times x_{n+1}\right)⋂\left|x_{n}-x_{n+1}\right|\geq 0\right],\;sgn\left(x\right)=\left\{\begin{array}{cc} 1, & x\geq threshold\\ 0, & otherwise \end{array}\right.$$

The slope sign attribute considers three adjacent samples to determine the number of times that a slope sign between these sEMG values changes:

(9) $$\mathrm{SSC}=\sum_{n=2}^{N}f\left(\left(x_{n}-x_{n-1}\right)\times \left(x_{n}-x_{n+1}\right)\right),\;f\left(x\right)=\left\{\begin{array}{cc} 1, & f=th\\ 0, & otherwise \end{array}\right.$$

where th is a threshold.

The quantity of motor unit action potential is estimated through the Willison amplitude by counting the number of times that two adjacent samples overcome a threshold, reducing artifacts produced by noise:

(10) $$\mathrm{WAMP}=\frac{1}{N}\sum_{n=1}^{N}f(|x_{n}\left|\right),\;f\left(x\right)=\left\{\begin{array}{cc} 1, & x\geq th\\ 0, & otherwise \end{array}\right.$$

The amount of muscular pulses is described by the log detector, which uses a threshold to avoid noisy samples. 

(11) $$\mathrm{MYOP}=\frac{1}{N}\sum_{n=1}^{N}f(|x_{n}\left|\right),\;f\left(x\right)=\left\{\begin{array}{cc} 1, & x\geq th\\ 0, & otherwise \end{array}\right.$$

### 2.3. Autoregressive Model Features

A linear autoregressive model describes a random process using *p* coefficients [37]. The goal consists of extracting *p* coefficients to construct a representation of each sEMG sample x[n] with the preceding signal values (x[n−1],x[n−2], …, x[n−p]), making a linear combination, which carries an error or white noise term:

(12) $$x\left[n\right]=\sum_{k=1}^{p}a_{k}x\left[n-k\right]+e\left[n\right]$$

where x[n] is the generated sEMG value through *k* earlier samples x[n−k], *p* is the order of the model, e[n] expresses an added error or white noise term, and $a_{k}$ is the autoregressive coefficients. The mathematical approach used to derive the autoregressive coefficients defines the regressive model type. The most popular autoregressive model is the Yule–Walker model, which uses the estimated values of the correlation function calculated as:

(13) $${\hat{r}}_{xx}\left(n,n-k\right)=Ex(n)x(n-k)k=0,\pm 1,\pm 2,\ldots$$

Having ${\hat{r}}_{xx}$ estimated with (13), a N×N squared Yule–Walker matrix equation is built as follows:

(14) $$\left[\begin{array}{cccc} {\hat{r}}_{xx}\left(0\right) & {\hat{r}}_{xx}\left(-1\right) & \cdots & {\hat{r}}_{xx}\left(-p\right)\\ {\hat{r}}_{xx}\left(1\right) & {\hat{r}}_{xx}\left(0\right) & \cdots & {\hat{r}}_{xx}\left(-p+1\right)\\ \vdots & \vdots & \cdots & \vdots \\ {\hat{r}}_{xx}\left(p\right) & {\hat{r}}_{xx}\left(p-1\right) & \vdots & {\hat{r}}_{xx}\left(0\right) \end{array}\right]\left[\begin{array}{c} 1\\ a_{1}\\ \vdots \\ a_{p} \end{array}\right]=\;\left[\begin{array}{c} \sigma_{w}^{2}\\ {\hat{r}}_{xx}\left(1\right)\\ \vdots \\ {\hat{r}}_{xx}\left(p\right) \end{array}\right]$$

where $\sigma_{w}^{2}$ is the variance of the modeled stochastic process. As the correlation matrix describes an equation system and fulfills the Toeplitz definition, the method uses the recursive Levinson–Durbin algorithm to get the autoregressive coefficients $a_{p}$.

Following a different approach, the Burg maximal entropy method, in [37,38], proposes the expansion of ${\hat{r}}_{xx}$, adding ${\hat{r}}_{xx}\left(p+1\right)$, ${\hat{r}}_{xx}\left(p+2\right)$, ${\hat{r}}_{xx}\left(p+3\right)$,… With this consideration in mind, the method extrapolates the new correlation values, maximizing the entropy between them, so that their randomness is high. The extrapolation of autoregressive series changes the predictions of backward and forward signal values $\hat{x}\left(n\right)$ and $\hat{x}\left(n-m\right)$:

(15) $$\hat{x}\left(n\right)=\sum_{k=1}^{m}a_{m}\left(k\right)x\left[n-k\right],0\leq k\leq m-1,m=1,2,\ldots p.$$

(16) $$\hat{x}\left(n-m\right)=-\sum_{k=1}^{m}{\left(a_{m}\right)}^{*}\left(k\right)x\left(n+k-m\right)0\leq k\leq m-1,m=1,2,\ldots ,p.$$

where $a_{m}\left(k\right)$ is the *k*^th^ autocorrelation coefficient of the model of order *m*, which implies a combination of previous values and the reflection coefficients $K_{m}$ [37]:

(17) $$a_{m}\left(k\right)=a_{m-1}\left(k\right)+k_{m}{\left(a_{m-1}\right)}^{*}\left(m-k\right),1\leq k\leq m-1,1\leq m\leq p.$$

The Burg proposal produces good results for different distributions; when the stochastic process has a Gaussian distribution, both autoregressive methods yield the same coefficient values [37].

### 2.4. Dataset Construction

**Figure 2:**
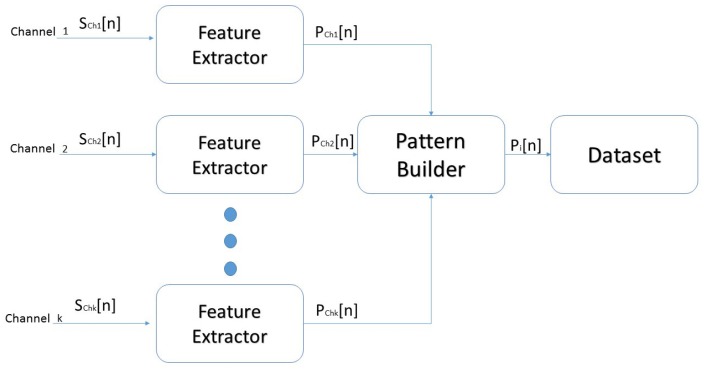
Dataset building block diagram.

To develop and test the proposed approach for hand movement classification, the features described above were first extracted from different channels $S_{Ch1}\left[n\right]$, …, $S_{Chk}\left[n\right]$ and placed in a dataset. Each feature extractor (see Figure 2) formed a single or multiple features, and its output was a vector that represented a pattern of the form $P_{Ch_{k}}\left[n\right]=[feature_{1}chk,feature_{2}chk$, …, $feature_{N}chk]$. Next, the derived features from the channels were transferred to the pattern builder, which concatenated the instances to generate an object containing the extracted features, and a label was assigned for the class instances $P_{i}\left[n\right]=[P_{Ch1}\left[n\right],P_{Ch2}\left[n\right]$, …, $P_{Chk}\left[n\right],class_{label}]$. Figure 2 shows the dataset building block diagram.

### 2.5. Proposed Classification Methodology by Applying Burg Reflection Coefficients

As mentioned in Section 2.3, the Burg autoregressive model introduces the forward and backward prediction errors:

(18) $$f_{m}\left(n\right)=x\left(n\right)-\hat{x}\left(n\right),b_{m}\left(n\right)=x\left(n-m\right)-\hat{x}\left(n-m\right).$$

These errors are defined by the following recursive renovation equations of the lattice linear prediction filter.

(19) $$\begin{array}{c} f_{0}\left(n\right)=b_{0}\left(n\right)=x\left(n\right),\\ f_{m}\left(n\right)=f_{m-1}\left(n\right)+K_{m}b_{m-1}\left(n-1\right)m=1,2,\ldots ,p,\\ b_{m}\left(n\right)=K_{m}^{*}f_{m-1}\left(n\right)+b_{m-1}\left(n-1\right)m=1,2,\ldots p. \end{array}$$

**Figure 3:**
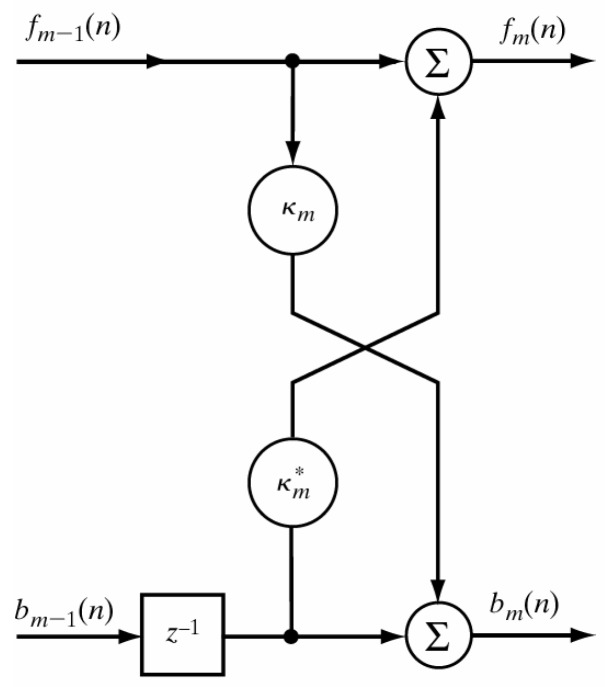
Lattice filter prediction cascade diagram.

Figure 3 illustrates how the recursive renewal equations are used to construct the linear lattice prediction filter. This modular structure simultaneously generates the prediction of forward and backward errors; and under certain conditions, the addition of modules decoupled from each other could increase the order of the filter. It has the advantage of simplifying the calculation, such as the storage of orthogonal variables of the output of $z^{-1}$ in each stage. The modular feature of the filter makes it suitable for its VLSI implementation.

The least squared error is:

(20) $$\varepsilon_{m}=\sum_{n=m}^{N-1}\left[\right|f_{m}{\left(n\right)|}^{2}+|b_{m}\left(n\right){|}^{2}].$$

Minimizing Expression (20), the reflection coefficients are obtained [37]:

(21) $$K_{m}^{*}=\frac{-\sum_{n=m+1}^{N-1}f_{m-1}\left(n\right)b_{m-1}^{*}\left(n-1\right)}{\frac{1}{2}\sum_{n=m+1}^{N-1}\left[\right|f_{m}\left(n\right)|t^{2}+|b_{m}\left(n\right){|}^{2}]},m=1,2,\ldots ,p.$$

Reflection coefficients are the harmonic mean value of backward and forward error coefficient cross-correlation. The numerator is the cross-correlation of the prediction errors, and the denominator is the smallest square estimation of these errors, so $|K_{m}|\leq 1$.

The information needed to compute the first *K* coefficient is obtained by developing the first expression in (19), so that for m=0:

(22) $$\begin{array}{c} f_{0}\left(0\right)=x\left(0\right),f_{0}\left(1\right)=x\left(1\right),\ldots ,f_{0}\left(n\right)=x\left(n\right)\\ b_{0}\left(0\right)=x\left(0\right),b_{0}\left(1\right)=x\left(1\right),\ldots ,b_{0}\left(n\right)=x\left(n\right). \end{array}$$

From this, it can be inferred that K${}_{1}$ is the autocorrelation normalized by signal energy, similar to the SSI value. This coefficient is used in the two remaining expressions in (19) with m=1 to obtain the necessary values to compute K${}_{2}$:

(23) $$\begin{array}{c} f_{1}\left(n\right)=f_{0}\left(n\right)+K_{1}b_{0}\left(n-1\right)=x\left(n\right)+K_{1}x\left(n-1\right)\\ b_{1}\left(n\right)=K_{1}^{*}f_{0}\left(n\right)+b_{0}\left(n-1\right)=K_{1}x\left(n\right)+x\left(n-1\right). \end{array}$$

This process continues with m=2, and so on, until the total amount of reflection coefficients is computed:

(24) $$\begin{array}{c} f_{2}\left(n\right)=f_{1}\left(n\right)+K_{2}b_{1}\left(n-1\right)=x\left(n\right)+K_{1}x\left(n-1\right)+K_{2}\left(K_{1}x\left(n-1\right)+x\left(n-2\right)\right)\\ b_{2}\left(n\right)=K_{2}^{*}f_{1}\left(n\right)+b_{1}\left(n-1\right)=K_{2}\left(x\left(n\right)+K_{1}x\left(n-1\right)\right)+K_{1}x\left(n-1\right)+x\left(n-2\right). \end{array}$$

The reflection coefficients (21) are computed iteratively through the signal values; this is the reason why they were proposed as features for classification tasks, as their complexity is linear, and there is no evidence of their usage in such tasks.

#### 2.5.1. Classification Model Training

Classification models involved in this research work were the Bayesian, *K* nearest neighbor, multilayer perceptron, decision trees, and support vector machines with different kernels models. These classifiers are available in the machine learning tool WEKA [39] and were chosen with the purpose of evaluating their performance using different sEMG features. For the training phase, the following three datasets were generated, comprised of 900 instances and 10 traits per channel:

- Time domain datasets (Equations (1)–(11)): TD= [IEMG MAV SSI VAR RMS WL WAMP SSC ZC MYOP]
- Burg autoregressive coefficients (Equation (17)): Arb = [Arb${}_{1}$ Arb${}_{2}$ … Arb${}_{n}$]
- Reflection coefficients: K = [K${}_{1}$ K${}_{2}$ … K${}_{n}$]

The classification algorithms were trained once, and the performance was obtained by K-fold cross-validation with a K value of 10, because it has been widely used in related state-of-the-art works and the datasets lacked class unbalance. Moreover, each instance took part in the training and testing set for a single run of the learning algorithm. Burg autoregressive coefficients (17) were chosen instead of Yule–Walker autoregressive coefficients (14) because different distributions of the Burg model produced a more accurate approximation [37]. After classifying the three main datasets, the K, Arb, and TD features were joined into a new dataset with patterns of the form of X = [IEMG MAV SSI VAR RMS WL WAMP SSC ZC MYOP Arb${}_{1}$ Arb${}_{2}$ … Arb${}_{n}$ K${}_{1}$ K${}_{2}$ … K${}_{n}$], in order to evaluate how the interaction between these different features was reflected in classification model performance.

#### 2.5.2. Features Selection and Reduction Methods

A high dimensionality feature vector sometimes implies having redundant or irrelevant traits that can affect the model training step because it is harder to find classification boundaries in a large dimensionality space; therefore, the output model would have a low performance. This phenomenon is known as “the curse of dimensionality”, and it is commonly faced in some feature selection methods.

Feature Selection guarantees a reduction in dimensionality with or without degradation of the classifier model’s performance. As observed in Section 2.2, some time domain features rely on the sEMG amplitude, such as RMS and MAV, and others depend on the number of occurrences of a certain event according to a threshold value. Burg autoregressive coefficients and reflection coefficients are relatedaccording to Equation (17). From the previous subsection, considering all the traits for the construction of the dataset *X*, it reaches a dimensionality of 60. There is a possibility that some of these traits are redundant or irrelevant and that the dimensionality space can be reduced by applying feature selection techniques.

Principal component analysis [40] produces new features based on a linear combination of the original characteristics. The new feature vectors consist of uncorrelated traits, the leftmost having the most variance in the dataset.

Subset Evaluation generates an original trait subset whose features are considered the most relevant because they are highly correlated with the class and have low intercorrelation [40]. The resulting set is the result of a search in the space state of the attribute subset by assessing the predictive ability of each feature individually and the degree of redundancy among them.

“Plus l-Take Away r” approach [41] is based on taking *l* traits and removing the remaining *r* features in such way that the classification performance remains high. If the exclusion of an attribute causes a lower performance than achieved prior, the removed sEMG characteristic is returned to the feature vector because it is deemed helpful in class instance assignment. This process is repeated until the dimensionality cannot be reduced without affecting the classification rate. Table 3 shows the result of applying this technique iteratively to dataset X; per channel, starting with all traits:

(25) $$l=30,r=0,[\mathrm{IEMG},\mathrm{MAV},\mathrm{SSI},\mathrm{VAR},\mathrm{RMS},\mathrm{WL},\mathrm{WAMP},\mathrm{SSC},\mathrm{ZC},\mathrm{MYOP},{\mathrm{Arb}}_{1}:{\mathrm{Arb}}_{10},K_{1}:K_{10}]$$

Feature Reduction process start by finding redundant time domain energy and counting event-related attributes, followed by Burg autoregressive and reflection coefficients. The reference classification performance is the highest when obtained by the instance-based classifier (IBk) in this case study.

From Table 3, at the first iteration, *r* increases by one and *l* decreases by the same rate, so that IEMG was removed; through Iteration 2–3, MAV and SSI were deleted. After this algorithm was repeated 20 times, removing any remaining attribute in the last row implied that the desired performance decreased; therefore, the removal process was stopped.

The resulting dataset was built from 10 features per channel, and 20 attributes were removed because they were not useful to build and maintain the performance of the IBk model.

Forward Selection method starts with an empty feature set, and attributes are added in such a way that the classification performance increases [40]. If adding a characteristic does not improve the model performance, it must be removed. The feature selection using this approach started taking the first Burg reflection coefficient per channel, and then, the first Burg autoregressive coefficient was added. Since the dataset built with $K_{1}$ and $Arb_{1}$ showed a high accuracy rate, it was taken as a basis, and the forward selection kept on choosing one of each energy and counting event-related trait among MAV, RMS, MYOP, and ZC, since they were considered the most relevant sEMG characteristics.

## 3. Results

Different classification models were trained with TD, Arb, K, and a combination of these datasets. In the experiments, the implementation of the classifiers in WEKA software was done. The classification results were evaluated in terms of the classification accuracy rate:

(26) $$R=\frac{TP+TN}{P}\cdot 100$$

where TP are true positives, TN are true negatives, and *P* denotes the total population.

### 3.1. Classification

This section provides the results of the classification of the movement of the hand with separate datasets. The modified parameter in the SVM model was the kernel function. For the rest of the models, the default WEKA parameters were not modified. Two values chosen for *k* in the IBk classifier were *k* = 1 and *k* = number for classes +1; assigning *k* = 6 would cause a tie among the six classes; the extra value will establish the majority class. Table 4 describes the results obtained by classifying the hand movements with separate datasets.

One can observe from Table 4 that the TD dataset and the SVM with third order polynomial kernel (P3 column) gave better decision boundaries than other kernels. The radial kernel yielded the lowest performance with Bayesian models; whereas the remaining models (trees and MLP) reached high classification rates; IBk with k=1 obtained the highest.

**Figure 4:**
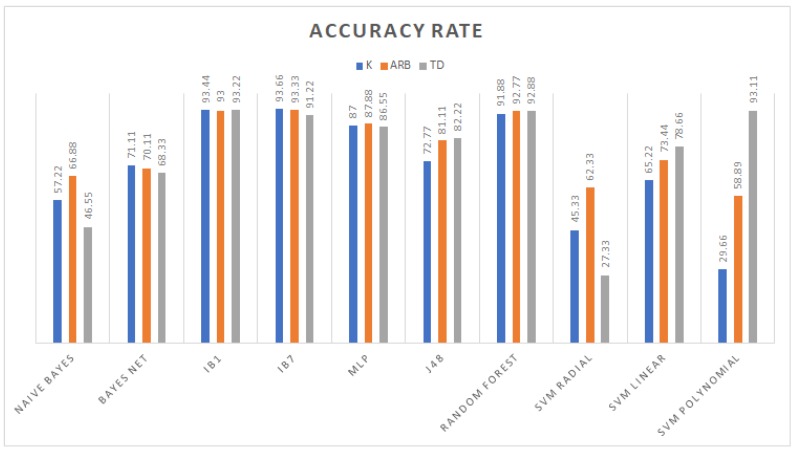
Accuracy rate achieved by the different classifiers.

**Figure 5:**
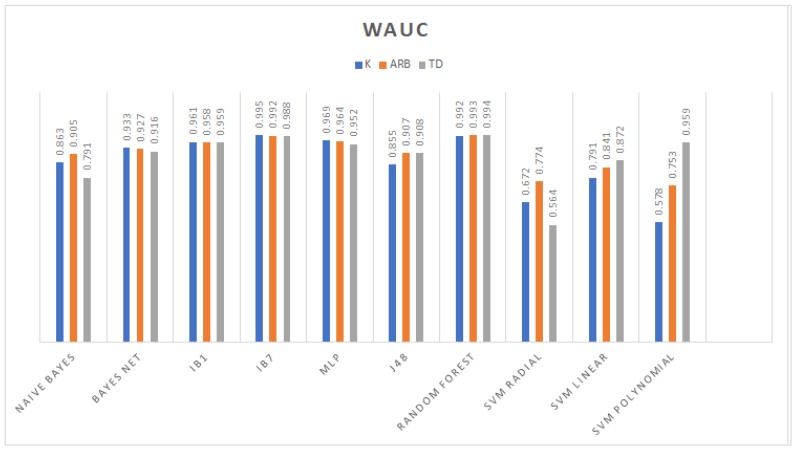
WAUC achieved by the different classifiers.

**Figure 6:**
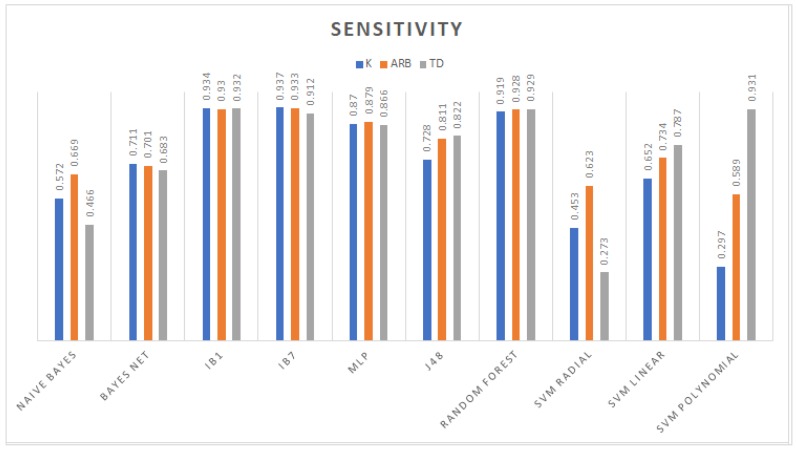
Sensitivity achieved by the different classifiers.

**Figure 7:**
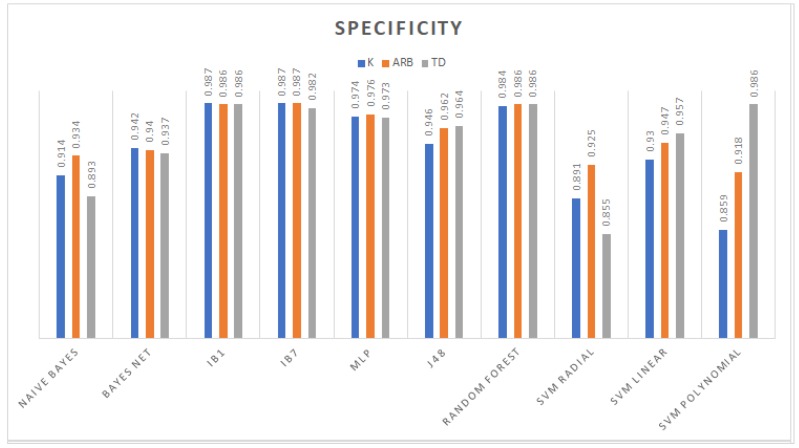
Specificity achieved by the different classifiers.

From the accuracy rate graph in Figure 4, the third order polynomial and linear kernel SVM trained with the TD dataset gave better decision boundaries than the radial kernel and other datasets. Furthermore, the J48 which is an algorithm used to generate a C4.5 decision tree developed by Ross Quinlan [39] and random forest models obtained higher performance compared to those generated using *K* and Arb. In particular, with this dataset, the radial kernel SVM yielded the lowest performance in conjunction with the Bayesian models; on the other hand, the remaining models (IBk and MLP) reached competitive classification rates. The evaluation metrics in the remaining graph among models trained with TD features show that random forest had the highest weighted area under the ROC curve (WAUC); IB1 had the biggest sensitivity; and regarding specificity, IB1, random forest, and polynomial SVM obtained the same value of 0.986. Figure 5, Figure 6 and Figure 7 show WAUC, sensitivity, and specificity.

IBk was more appropriate to classify the dataset built only with maximal entropy autoregressive coefficients (Arb column) considering seven neighbors more than just one. Other learning algorithms such as MLP and decision trees offered competitive classification rates (78–90.77%); Bayesian models outperformed linear kernel support vector machines with a performance of 63.33%. The best performance among the different datasets was obtained using the reflection coefficient dataset *K* of the reflection coefficients, classifying 93.55% of instances using IBk k=1. Despite Bayesian models and SVM still having a low performance, an improvement in the Bayesian net was achieved using the reflection coefficients.

In Table 4, by merging reflection coefficients and TD features for the training phase (K + TD), most of the classifiers reached a high performance, excluding naive Bayes and radial kernel SVM. With a 0.22% classification error, IBk k=1 obtained the best classification rate of the following models: IBk k=7, MLP, decision trees, and third orderpolynomial kernel SVM. The resulting dataset of joining the Burg maximal entropy reflection coefficients, *K*, and the Burg autoregressive coefficients, Arb, yielded patterns that were best classified by the IBk model with a *k* value of one, slightly above the IBk using k=7, MLP, and random forest. The J48 decision tree and Bayesian models offered high performances and were below the 90% accuracy reached by linear kernel SVM; the other kernels had the lowest classification rates. The combination of all features (TD, Arb, and K) resulted in a sixty-dimension feature vector, useful to classify all 900 dataset instances correctly using the IBk with k=1; increasing the number of neighbors to k=7 decreased the classification rate, but remained above the following competitive models: MLP and random forest. The data distribution did not fit to a radial kernel; therefore, the SVM output the lowest accuracy.

### 3.2. Feature Selection Classification Performance

This subsection describes the results of dimensionality reduction trying to reach a higher classification performance with as few traits as possible. After running the feature selection algorithms, all instances continued to be classified correctly with 20 and 26 features with the nearest neighbors and support vector machine models, respectively (see Table 5).

SE traits exclude amplitude-related values such as RMS, MAV, and so on. They provided high classification rates with the exception of the linear kernel SVM. The PC dataset was built with the combination of the less correlated features, resulting in a more uniform performance through all tested classifiers, reaching a 100% performance rate using the SVM with a radial kernel. The $FS_{1}$ represented the result of the feature selection process; the IBk with *k* = 1 was still the highest and SVM with any kernel the lowest in performance. Datasets $FS_{2}$, $FS_{3}$, $FS_{4}$, and $FS_{5}$ were obtained taking the first reflection and autoregressive coefficients in combination with MYOP, ZCC, RMS, and MAV. These sets had low pattern dimensionality and had high performance, from 83% up to 99.22% using the IBk with k=1 (the highest), MLP, decision trees, and Bayes net (the lowest); the remaining models yielded a low performance with just eight attributes.

## 4. Discussion

The classification accuracy rate of different learning algorithms depends on the data distribution. This behavior is expected according to the “no-free-lunch theorems” [42], which state that the best classification model for all datasets does not exist. The justification of why several models have to be compared using the same dataset concurs with that statement.

While testing classifiers with separate datasets, Burg reflection coefficients *K* (21) are more appropriate to use in conjunction with the Bayes net and IBk models. Since *K* traits are needed to compute the Burg autoregressive traits, Arb (17), their classification performances are similar. Besides, TD features have different values, which rely on amplitude or counting events. Therefore, the MLP, decision trees, and linear and polynomial kernel SVM outputs have high accuracy.

Despite the Burg maximal entropy autoregressive and reflection coefficients being closely related, the classification rate increases when these two traits take a part of the dataset. This means that they are not redundant or irrelevant in feature construction tasks; however, as mentioned before, Arb characteristics take more time to compute because they rely on first computing the reflection coefficients. In addition, as a result of combining three different theoretical frameworks’ attributes, the highest classification rate was obtained. Hence, a synergy of different attributes is needed for a higher accuracy in the sEMG classification tasks.

As it is shown in Table 6 compared against the results shown in Table 2, a high classification performance was reached with 26 or fewer features. The fact that the feature vector *X* had at least 34 redundant or irrelevant traits, which needed to be removed using feature selection tools, can explain this. As a result, different data distributions were obtained; for instance, principal component analysis performed by WEKA produced a data distribution suitable for support vector machine with a radial kernel since all patterns were correctly classified.

Excluding the PC dataset, the best classifier for the remaining datasets was the IBk model with a different number of neighbors used for class assignment, because the performance of the nearest neighbors-based model depended on the value of *k*. For instance, the TD and *K* dataset classification rates decreased as a result of increasing the value of *k*, contrasting what happened with the Arb patterns (see Table 5). This behavior is expected because the classification phase applies the nearest neighbor to different classes, causing in the worst case a tie and a misclassified pattern.

The advantage of IBk is its simple training phase, which is based directly on the dataset, compared to the Bayesian models, which require the computation of probability distributions and the cost function. The design of the hidden layer of MLP can be complex, and the time for reducing back propagation error might be long. The decision trees, as well as multilayer perceptron are hard to design, and they require a pruning process to reduce irrelevant leaves and branches, while SVM has a complexity of $O(n_{3})$ to establish support vectors. The kernel selection and design of the support vectors that best fit the data distribution are needed.

An example of kernel selection can be seen from Table 5 with the TD dataset using the polynomial kernel, a high performance of 93.11% was reached, and merging the TD with *K* features, an improvement of 0.11% was achieved. This is a sign that most of the support vectors were found in the TD traits using such a kernel. Another example is comprised of the Arb and *K* traits: they were better classified with a linear kernel, and by joining these two datasets, a considerable increase in the classification rate was obtained (see Table 5) as a consequence of more appropriate data for the support vector estimation.

The results that are considered for the discussion are those that were obtained using the same signal dataset; otherwise, any comparison concerning the feature extraction and preprocessing stage based on the classification performance would be unfair.

The feature extraction method presented in [29] yielded fifty traits and reached a high performance (98.22%) with no previous signal treatment, while the classification rate of 89.21% was obtained with the empirical mode decomposition technique, which denoised the original signal in conjunction with the sixty four features proposed in [30]. Our methodology succeeded in classifying all 900 sEMG instances with less than half the features (20 characteristics) as in the methods of [29,30]. Their results were surpassed with only eight traits, producing 99.22% correctly-classified signals. Our preprocessing stage consisted only of the reduction of noisy samples and the subtraction of the myoelectric signal mean value, which turned out to be effective because it gave signals suitable for the framework of the developed feature extraction tools, such as the Burg reflection coefficients (21).

As shown in [30], decomposing sEMG caused information loss, which was reflected in the classification rate; for this reason, all available signal information for the feature extraction task was taken.

## 5. Conclusions

The results of hand movement classification of myoelectric signals using different traits have been presented. The Burg linear autoregressive model yielded the reflection coefficients that were shown to be useful for sEMG classification; these traits showed a higher performance that was increased with the standard time domain features and was less complex to compute than the autoregressive coefficients.

A high classification rate was reached using a simple preprocessing stage, which fit the signals to the theoretical framework in the feature extraction tools. Low pattern dimensionality resulted from removing redundant traits, building patterns with uncorrelated features such as Burg maximal entropy reflection and autoregressive coefficients, and considering different time domain features related to the signal energy and to event counting. Despite a lower feature dimensionality, a classification rate of up to 100% has been achieved separately and with other kinds of uncorrelated attributes for the hand movement identification.

The applications of the classification technique for signals presented in this work can lead to the possible development of state-of-the-art active prosthetic devices, where the myoelectric classification does not represent a challenge and the implementation of the classification algorithm can be performed in an integrated low-cost device.

## Figures and Tables

**Table 1:** Time-domain and frequency-domain features used in sEMG data processing and classification tasks.

	Feature	Abbreviation
1	Root mean squared value	RMS
2	Mean average value	MAV
3	Variance	VAR
4	Willison amplitude	WAMP
5	Wavelength	WL
6	Auto-regressive	AR
7	Difference absolute mean value	DAMV
8	Difference absolute standard deviation value	DASDV
9	Difference absolute variance	DVARV
10	Difference absolute standard deviation	DASDV
11	Second order moment	M2
12	Integrated EMG	IEMG
13	Simple squared integration	SSI
14	Myopulse percentage rate	MYOP
15	Cepstral coefficients	CC
16	Log detector	LOG
17	Temporal moment	TK
18	V order	V
19	Zero crossings	ZC
20	Slope sign change	SSC

**Table 2:** Feature extraction techniques for hand movement classification applied to the EMG dataset from the University of California at Irvine (UCI) machine learning repository.

Research Group	Algorithm	Accuracy
[32]	Neural Network after Empirical Mode Decomposition (EDM)	85%
[32]	Adaptive Boosting after EMD	55%
[32]	Linear Discriminant Analysis after EMD	65%
[32]	Random Forest after EMD	91%
[32]	Random Forest + PCA after EMD	94%
[29]	Singular-Value Decomposition with SVM	98.22%
[29]	k-Nearest Neighbor	94.77%
[29]	Naive Bayes	91.66%
[29]	Radial Basis Function Network	94%

**Table 3:** Feature reduction process. MAV, mean average value; SSI, simple squared integration; WL, wavelength; WAMP, Willison amplitude; MYOP, myopulse percentage rate.

l	r	Remaining Features
29	1	[MAV, SSI, VAR, RMS, WL, WAMP, SSC, ZC, MYOP, Arb${}_{1}$:Arb${}_{10}$, K${}_{1}$:K${}_{10}$]
28	2	[SSI, VAR, RMS, WL, WAMP, SSC, ZC, MYOP, Arb${}_{1}$:Arb${}_{10}$, K${}_{1}$:K${}_{10}$
27	3	[VAR, RMS, WL, WAMP, SSC, ZC, MYOP, Arb${}_{1}$:Arb${}_{10}$, K${}_{1}$:K${}_{10}$]
⋮	⋮	⋮
10	20	[ZC, MYOP, Arb${}_{1}$, Arb${}_{2}$, Arb${}_{7}$, Arb${}_{8}$, Arb${}_{10}$, K${}_{1}$, K${}_{2}$, K${}_{10}$]

**Table 4:** Classification results of the time domain (TD), Arb, and *K* datasets separately. P3, third order polynomial.

N	Dataset	Bayes		IBk		MLP		Tree		SVM	
−	−	Naive	net	$k_{1}$	$k_{7}$	−	J48	Random	radial	linear	P3
20	TD	46.55	68.33	93.22	91.22	86.55	83.33	92.88	27.33	76.33	93.11
20	Arb	59.11	63.33	90.77	91.66	86.33	78.00	90.55	47.00	61.11	37.77
20	k	57.44	71.11	93.55	93.33	86.44	74.77	92.00	45.55	65.44	29.77

**Table 5:** Classification performance of the combined datasets.

N	Features	Bayes		IBk		MLP		Tree		SVM	
−	−	Naive	net	$k_{1}$	$k_{7}$	−	J48	Random	radial	linear	P3
40	k+TD	61.44	83.22	99.88	99.55	98.44	87.11	98.33	18.33	83.11	93.22
40	k+Arb	78.22	82.33	99.66	99.33	98.44	84.77	98.66	64.00	90.00	54.66
60	*X*	76.00	83.33	100.0	99.77	99.11	85.55	95.55	17.77	83.00	93.22

**Table 6:** Classification performances using different feature vectors after feature selection: SE = [$Ch_{1}$Arb(1,2,5,9,10), $ch_{1}$k(1,2,3,4,9), $Ch_{1}$WL, $Ch_{1}$SSC, $Ch_{1}$ZC, $Ch_{1}$MYOP, $Ch_{2}$Arb(1,2,4,5,10), $Ch_{2}k$(1,5,7), $Ch_{2}$WL, $Ch_{2}$MYOP], $FS_{1}$ = [Arb(1,2,7,8,10), K(1,2,10), ZCC, MYOP], $FS_{2}$ = [Arb1, K1, ZCC, RMS], $FS_{3}$ = [$Arb_{1}$, $K_{1}$, ZCC, MAV], $FS_{4}$ = [$Arb_{1}$, $K_{1}$, MYOP, RMS] $FS_{5}$ = [$Arb_{1}$, $K_{1}$, MYOP, MAV].

N	Features	Bayes		IBk		MLP		Tree		SVM	
−	−	Naive	net	$k_{1}$	$k_{7}$	−	J48	Random	radial	linear	P3
26	SE	82.55	91.55	99.88	99.88	99.11	87.77	99.55	18.00	74.33	82.00
26	PC	88.11	88.66	99.66	98.88	97.55	84.11	98.44	100.0	96.11	99.55
20	$FS_{1}$	79.88	86.88	100.0	99.66	97.66	86.33	98.77	24.88	68.88	57.77
8	$FS_{2}$	64.88	84.22	99.22	99.22	88.55	88.66	97.88	22.33	55.11	49.88
8	$FS_{3}$	65.00	83.22	99.22	98.77	89.22	89.55	97.88	22.33	54.33	45.88
8	$FS_{4}$	66.77	84.66	98.22	97.77	87.55	88.77	89.88	66.11	70.77	28.66
8	$FS_{5}$	67.33	83.00	98.22	97.77	88.55	88.55	98.33	61.44	68.66	32.55
